# Editorial: Hazardous substances from food processing: Formation and control, biotoxicity and mitigation

**DOI:** 10.3389/fnut.2022.1118936

**Published:** 2022-12-20

**Authors:** Iftikhar Ali Khan, Chong Wang, Kezhou Cai

**Affiliations:** ^1^Shenzhen Key Laboratory of Marine Microbiome Engineering, Institute for Advanced Study, Shenzhen University, Shenzhen, China; ^2^College of Food Science and Technology, Nanjing Agricultural University, Nanjing, China; ^3^School of Food and Biological Engineering, Hefei University of Technology, Hefei, China

**Keywords:** food processing, hazardous substances, occurrence, toxicity, health risk, inhibition

The Maillard reaction produces flavor and aroma compounds in thermally processed and baked foods. Unfortunately, their formation is frequently accompanied by undesirable reactions that can result in various hazardous Maillard products, such as heterocyclic amines (HCAs), acrylamide (AA), furan, and lipid and protein oxidation products. Furthermore, oxidation of unsaturated fatty acids (UFAs) present in plant-based oil during thermal processing results in the production of several radicals, such as •CH3, •CO, and •CHO, which may then produce malondialdehyde (MDA), aldehydes, volatile oxidation products and α-dicarbonyl compounds, such as glyoxal (GO), and methylglyoxal (MGO) (1, 2). Furthermore, GO, and MGO serves as a precursor for acrylamide and advanced glycation end products (AGEs) (3). Even though these hazardous substances/compounds are generally found at very low concentrations (ppb), animal studies have revealed that many are carcinogenic and have significant mutagenicity in bacterial assays (4).

Moreover, epidemiological studies show a positive association between these hazardous compounds and the incidence of cancers such as stomach, breast, intestinal, and colorectal cancer (5). These toxic substances can be produced in industrial food processes and during ordinary household cooking, as both high temperatures (>150 °C) and prolonged heating promote their formation. In other words, avoiding dietary exposure to them is nearly impossible for the general public. As a result, they may pose serious health risks, and inhibiting their formation has been a long-term strategy for developing safe and healthy foods. This important editorial initiative focused on the formation, occurrence, analysis methods, toxicological and health aspects, and control of various hazardous substances/compounds in processed and baked foods. The purpose of this special issue Research Topic was to provide a comprehensive overview of the formation mechanism, control methods, and biological toxicity, with a special emphasis on inhibition of hazardous food substances and future challenges, to provide an extensive overview of the field and to identify novel research areas to ensure food safety and human health.

Zhuang et al. aimed to examine the effects of fatty acid type and heating temperature on the formation of lipid oxidation products: malondialdehyde (MDA), α-dicarbonyl compounds, volatile aldehydes, and unsaturated aldehydes in vegetable oils and concluded that potential risk to human health could rise as a result of high temperature and the loss of unsaturated fatty acids, which could cause an increase in hazardous lipid oxidation products.

Kim et al. investigated the level of furan in dried red pepper powder after boiling, roasting, and frying, as well as the kinetics of furan in roasted dried red pepper powder and the effect of the fatty acid composition of the oil in which the powder was cooked, was explored.

Ahmed et al. explored how baking and frying conditions affect acrylamide formation in locally produced fried and baked products in Pakistan. They concluded that the highest level of acrylamide was found in paratha roll, followed by potato cutlets and biscuits. Furthermore, baking and frying at higher temperatures resulted in higher acrylamide formation in these products.

Rao et al. identified the predominant lipid compounds in sugarcane and emphasized their unique pharmacological value. They discovered that 2- linoleoylglycerol and gingerglycolipid C have potent binding interactions with the 3CL^pro^ of SARS-CoV-2 and that these compounds could be used as SARS-CoV-2 therapeutic agents.

The work by Khan et al. evaluated the impact of replacing chicken fat with Perilla seed meal (PSM) on lipid and protein oxidation, fatty acid profile, volatile flavor compounds, and carcinogenic heterocyclic amines (HCAs) in pan-fried low-fat chicken patties. The results of this study not only endorse the tremendous potential of PSM for use as a fat substitute to improve the fatty acid profile and decrease the content of harmful byproducts in heat-processed chicken but also emphasize the significance of incorporating the appropriate level of PSM to maximize their functional potential.

In conclusion, we believe that this compilation of articles will provide sufficient information to the readers and inspire and motivate young researchers and authors working in the area of food chemistry and toxicology.

## Author contributions

IK wrote the initial draft of the manuscript. CW and KC finalized the manuscript. All authors contributed to the article and approved the submitted version.
